# Effectiveness and Safety of Immune Checkpoint Inhibitors in Colorectal Cancer: A Systematic Review of Real-World Studies

**DOI:** 10.1007/s11912-025-01676-0

**Published:** 2025-05-13

**Authors:** Leping Kong, Chin Hang Yiu, Christine Y. Lu

**Affiliations:** 1 The University of Sydney School of Pharmacy, Camperdown, NSW Australia; 2 Kolling Institute, Faculty of Medicine and Health, The University of Sydney and the Northern Sydney Local Health District, Sydney, NSW Australia; 3 Department of Pharmacy, Royal North Shore Hospital, St Leonards, New South Wales Australia

**Keywords:** Colorectal Cancer, Immune Checkpoint Inhibitors, Real-World Evidence, Microsatellite Instability, Survival Outcomes, Safety Outcomes

## Abstract

**Purpose of review:**

Immune checkpoint inhibitors (ICIs) have demonstrated significant efficacy in the treatment of colorectal cancer (CRC). However, most evidence has come from clinical trials with strict eligibility criteria. Understanding real-world effectiveness and safety of ICIs in CRC is important to guide routine clinical practice across diverse populations.

**Recent findings:**

A systematic review following PRISMA guidelines was conducted to identify observational studies evaluating ICI-based regimens compared to conventional or combination therapies in patients with CRC. Three databases (MEDLINE, Embase, and Scopus) were searched from inception through March 15, 2025. Eligible studies reported at least one efficacy outcome (e.g., progression-free survival [PFS], overall survival [OS], etc.) and/or safety outcome (e.g., adverse events) among real-world populations with CRC treated with ICIs. Study quality was assessed using the Newcastle–Ottawa Scale, and a narrative synthesis was performed to summarize the key findings.

Eleven real-world studies met the inclusion criteria, encompassing data from 2,049 patients. In MSI-H/dMMR metastatic CRC, real-world findings aligned with the survival benefits observed in clinical trials, demonstrating improved PFS and OS compared to conventional therapies. For MSS/pMMR metastatic CRC, combining ICIs with other agents (e.g., tyrosine kinase inhibitors or chemotherapy) showed improvements but yielded conflicting results. Overall, the safety profiles were comparable to conventional therapies, with treatment-related adverse events occurring at similar rates.

**Summary:**

Real-world evidence supports the efficacy of ICI monotherapy in MSI-H/dMMR metastatic CRC and suggests potential benefits of ICI-based combination therapies in MSS/pMMR metastatic CRC. However, most of the data are derived from small, single-center cohorts, which limit their generalizability. Further multi-center studies are needed, especially to assess the efficacy of ICI-based combination therapies in the broader CRC population.

**Supplementary Information:**

The online version contains supplementary material available at 10.1007/s11912-025-01676-0.

## Introduction

Colorectal cancer (CRC) is the third most commonly diagnosed cancer and the second leading cause of cancer-related deaths worldwide, accounting for 10% of all cancer cases and 9.4% of all cancer-related deaths [1]. Despite advances in detection, over 20% of CRC patients are diagnosed at the metastatic stage, which complicates treatment with chemotherapy and targeted therapies, such as those targeting vascular endothelial growth factor (VEGF) and epidermal growth factor receptor (EGFR) [2, 3].

Immune checkpoint inhibitors (ICIs) are a class of immunotherapy agents that work by modulating regulatory immune checkpoints, enabling the immune system to recognize and attack tumor cells [4]. ICIs targeting programmed cell death protein (PD- 1) and its ligand PD-L1 have demonstrated significant efficacy in tumors with high mutational burden and neo-antigen loads, including DNA-deficient mismatch repair (dMMR) and microsatellite instability-high (MSI-H) CRC [5, 6]. Recently published trial KEYNOTE- 177 have led to the inclusion of ICIs as a first-line treatment for MSI-H/dMMR metastatic CRC (mCRC) in the National Comprehensive Cancer Network guidelines [7]. These studies demonstrated superior objective response rates and median progression-free survival (PFS) with pembrolizumab compared to chemotherapy. Specifically, pembrolizumab achieved an objective response rate (ORR) of 45.1% and a median PFS of 16.5 months, versus 33.1% and 8.2 months with chemotherapy [7]. However, MSI-H/dMMR tumors account for only about 5% of all CRC cases [7, 8]. Most patients with mismatch repair-proficient (pMMR) or microsatellite stable (MSS) mCRC do not respond well to ICIs, especially when used as monotherapy [9–12]. To address this limitation, combining ICIs with other therapies, such as anti-angiogenic drugs or chemotherapy, has emerged as a potential strategy. For example, PD- 1 inhibitors combined with regorafenib showed promising efficacy (median PFS = 7.9 months, ORR = 36%) in the phase I REGONIVO trial, where 96% of patients had MSS CRC [13]. Another phase Ib/II trial, REGOTORI, involving 38 patients with MSS mCRC, showed a median PFS of 2.1 months and an ORR of 15.2% [14]. However, most studies to date have been phase I and single-arm designed, and data on the efficacy and safety of ICI combination therapies with standard treatments from phase III studies are still emerging.

Patients enrolled in randomized controlled trials (RCTs) often meet strict inclusion criteria, which may not fully represent the broader patient population seen in routine clinical practice. In real-world settings, factors such as comorbidities, concurrent medications used, and socioeconomic differences can influence treatment outcomes [15–17]. As a result, real-world data from sources like administrative claims, electronic health records and population-based cancer registries provides valuable insights that complement the controlled settings of clinical trials [15, 16, 18]. While existing systematic reviews and meta-analyses of ICIs using real-world data have primarily focused on lung cancer [19–21], no such reviews have been published specifically for CRC. By examining the use of ICIs in a broader, more heterogeneous CRC population, real-world studies can identify patterns of efficacy and safety that may not be captured in clinical trials. This systematic review aims to assess real-world evidence, with a focus on comparing ICI-based regimens to conventional treatments, to better inform clinical practice and guide future research.

## Methods

### Search Strategy

This systematic review follows the Preferred Reporting Items for Systematic Reviews and Meta-analyses (PRISMA) guidelines [22]. The protocol was prospectively registered with PROSPERO (ID: CRD42024575484). Changes made to the PROSPERO protocol, along with the corresponding reasons, are provided in Supplementary Table S1. A comprehensive search was conducted across three electronic databases—MEDLINE, Embase, and Scopus—covering studies from database inception through March 15, 2025. The full search strategy is provided in Supplementary Table S2.

### Eligibility Criteria

This review included observational studies reporting real-world outcomes of ICIs in CRC patients, with no limitations on the adjuvant or metastatic setting. Studies were eligible for inclusion if they included a comparison or control group (e.g., standard chemotherapy, targeted therapy) to evaluate the comparative effectiveness and safety of ICI-based regimens versus standard treatments. Eligible studies had to report at least one efficacy (e.g., response to treatment, overall survival) and/or safety outcome. Exclusion criteria included RCTs, commentaries, editorials, reviews, studies published in languages other than English, and conference or posters without full-text publication.

### Outcomes

Efficacy outcomes included PFS, overall survival (OS), ORR and disease control rate (DCR). Median PFS refers to the time at which 50% of patients have experienced tumor progression, while median OS is the time at which 50% of patients are still alive. ORR was defined as the proportion of patients with a confirmed complete or partial response, and DCR was the proportion of patients with a complete or partial response, or stable disease under ICIs. Safety outcomes included treatment-related adverse events (TRAEs) and treatment discontinuation rates.

### Study Selection

Duplicates were removed, and LK and CHY independently screened all identified titles and abstracts for eligibility using the Covidence platform (Veritas Health Innovation, Melbourne, Australia) [23]. Studies deemed relevant were then evaluated in full text to confirm eligibility. Disagreements were resolved through consensus or discussion with a senior investigator (CYL).

### Data Extraction

Two investigators (LK and CHY) independently extracted data from the included studies, and any disagreements were resolved through consensus or discussion with a third investigator (CYL). The following data were extracted: study characteristics (e.g., author, year), patient population, intervention groups, comparator groups, efficacy outcomes, and safety outcomes.

### Data Synthesis

A meta-analysis was not conducted due to the considerable variability in study populations, designs, outcome measures, and statistical approaches. Therefore, a descriptive synthesis of the key findings was conducted.

### Quality Assessment

The Newcastle–Ottawa Scale (NOS) was used to assess the quality of the included non-randomized, non-interventional cohort studies by two investigators (LK and CHY) [24]. Studies were evaluated based on three criteria: selection, comparability, and outcome assessment. The scoring system assigns up to 9 stars, with a maximum of 4 stars for selection, 2 stars for comparability, and 3 stars for outcome measures. Each study’s overall quality was then assessed using the Agency for Healthcare Research and Quality (AHRQ) criteria and classified as good, fair, or poor [25].

## Results

### Article Selection

**Fig. 1:**
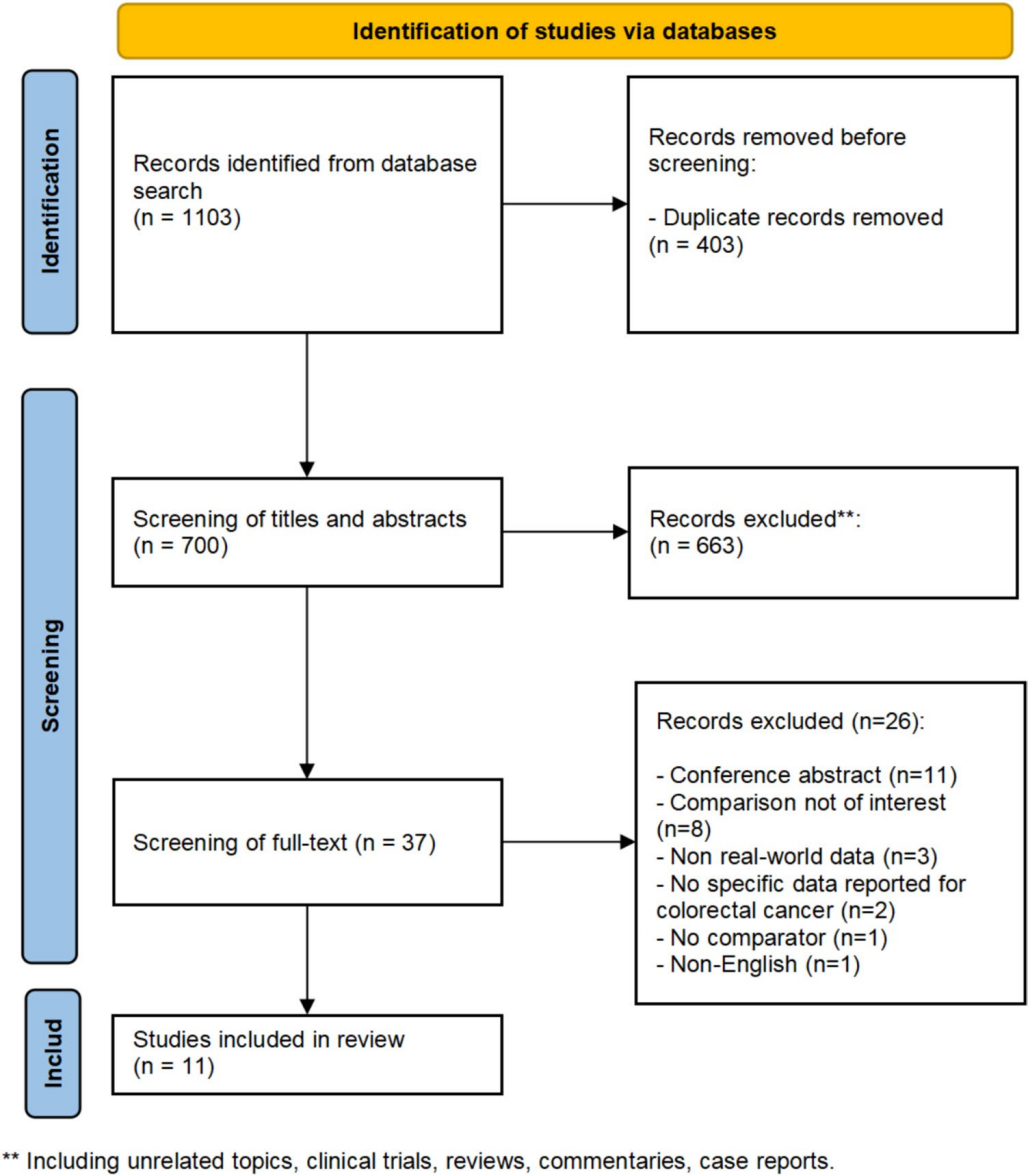
PRISMA flow diagram of the study selection process [22]

A total of 1,103 records were identified from the three databases. After removing the duplicates (n = 403), 663 records were excluded based on title and abstract screening. The full text of 37 records was then evaluated, and 11 observational studies that used real-world data (all based on electronic health records) were included in this review (Fig. 1) [26–36]. All 11 studies focused on mCRC patients. The majority of the studies were conducted in China (n = 10) [27–36], and one study was conducted in the United States [26]. Five studies did not report results stratified by MSI-H or MSS tumor type [27–30, 33], though MSS/pMMR comprised the majority of patient MSI status. Five studies were specifically conducted in the MSS mCRC setting [31, 32, 34–36]. One study included two cohorts: cohort one was specifically conducted in MSI-H mCRC setting, while cohort two included both MSI-H mCRC and MSS mCRC patients [26].

Table 1 summarizes the characteristics of the included studies [26–36]. Of those that reported, the median duration of follow-up ranged from 10.8 to 28.4 months. A total of 2,049 patients were included across all studies, with sample sizes varying from 60 to 537 patients (median = 129) [26–36]. The ICIs used in the studies included camrelizumab, nivolumab, pembrolizumab, penpulimab, sintilimab, tislelizumab and toripalimab [26–36]. Comparators included conventional chemotherapy and other medications. Adverse reactions were graded according to the National Cancer Institute Common Terminology Criteria for Adverse Events [37].

Among studies involving patients with MSS mCRC, nine included liver metastasis as a prognostic factor in their analyses [27, 29–36]. Of these, three studies performed stratified analysis based on liver metastases within ICI-based treatment groups [31, 35, 36]. An et al. reported that OS was significantly prolonged in patients without liver metastases who received ICI plus fruquintinib treatment [31]. Similarly, Zhao et al. found that patients without liver metastases had significantly longer median PFS (6.7 months vs 3.5 months, *p* < 0.0001) and median OS (15.8 months vs 10.6 months, *p* = 0.045) [36]. Additionally, in a study of 38 patients without liver metastases, those receiving ICI combination therapy had significantly longer median PFS compared to those in the non-ICI group (7.1 months vs 5.6 months, *p* = 0.034) [35].

**Table 1:** Characteristics of included studies (*n* = 11)

Author (Year)	Country	Experimental groups, sample size	Comparator groups, sample size	Efficacy data	Safety data
Quintanilha (2023)	United States	Cohort 1 involved patients receiving first-line ICIs ICIs (pembrolizumab, nivolumab, ipilimumab plus nivolumab), MSI-H CRC patients only, *n* = 49	Cohort 1: Chemotherapy, *n* = 89	TTNT: ICI: NR Chemotherapy: 7.23 months (IQR, 6.21–9.72 months; AHR, 0.17; 95% CI, 0.08–0.35; *p* < 0.001) mPFS: ICI: 24.87 months [IQR, 19.10 months to NR] Chemotherapy: 5.65 months (IQR, 4.70–8.34 months); AHR, 0.31 (95% CI: 0.18–0.52); *p* < 0.001) mOS: ICI: NR Chemotherapy: 24.1 months (IQR, 13.90 months to NR); AHR, 0.45 (95% CI: 0.23–0.88); *p* = 0.02)	None
		Cohort 2 involved patients receiving ICIs at any line of therapy MSI-H CRC patients receiving ICIs at any line of therapy (n = 83). ICIs (pembrolizumab, nivolumab, ipilimumab plus nivolumab) vs MSS CRC patients receiving the same ICIs at any line of therapy (n = 99)		TTNT: MSI-H CRC: NR MSS CRC: 6.64 months (IQR, 5.06 months – NR; AHR, 0.30; 95% CI, 0.17–0.54; *p* < 0.001) mPFS: MSI-H CRC: 12.19 months (IQR, 6.24–25.86 months) MSS CRC: 2.46 months (IQR, 1.97–2.79 months); AHR, 0.32; 95% CI, 0.21–0.47; *p* < 0.001) mOS: MSI-H CRC: NR MSS CRC: 6.57 months (IQR, 4.14–8.02 months); AHR, 0.25; 95% CI, 0.14–0.44; *p* < 0.001)	None
Nie et al. (2023)	China	ICIs (tislelizumab) + TAS- 102, *n* = 10	TAS- 102 Monotherapy, *n* = 6 TAS- 102 + Bevacizumab, *n* = 54	mPFS: ICI + TAS- 102: 3.0 months (95% CI: 1.5–4.5, *p* = 0.041) TAS- 102 Monotherapy: 3.0 months (95% CI: 2.2–3.8) TAS- 102 + Bevacizumab: 6.3 months (95% CI: 5.2–7.4) mOS: ICI + TAS- 102: 6.0 months (95% CI: 1.7–10.3, *p* = 0.013) TAS- 102 Monotherapy: 6.5 months (95% CI: 0.1–12.9) TAS- 102 + Bevacizumab: 12.0 months (95% CI: 9.6–14.4) ORR: ICI + TAS- 102: 0%, *p* = 0.860 TAS- 102 Monotherapy: 0% TAS- 102 + Bevacizumab: 1.9% DCR: ICI + TAS- 102: 40%, *p* = 0.047 TAS- 102 Monotherapy: 50% TAS- 102 + Bevacizumab: 75.9%	The combination of ICI + TAS- 102 and TAS- 102 + bevacizumab did not significantly increase the incidence of TAS- 102-related adverse events
An et al. (2024)	China	PD- 1 inhibitors (camrelizumab, sintilimab, tislelizumab, toripalimab, pembrolizumab, nivolumab) + Fruquintinib, *n* = 95 PD- 1 inhibitors (camrelizumab, sintilimab, tislelizumab, toripalimab, pembrolizumab, nivolumab) + Regorafenib, *n* = 81	Fruquintinib, *n* = 70 Regorafenib, *n* = 67	mPFS: ICI + Fruquintinib: 4.9 (95%CI: 3.0–6.8) months Fruquintinib: 3.5 (95%CI: 2.9–4.1) months, *p* = 0.057 ICI + Regorafenib: 3.0 (95%CI: 2.0–4.1) months Regorafenib: 3.6 (95%CI: 2.5–4.7) months, *p* = 0.534 6-month PFS: ICI + Fruquintinib: 47.1% Fruquintinib: 27.8%, *p* = 0.014, ICI + Regorafenib: 22.0% Regorafenib: 27.8%, *p* = 0.521 mOS: ICI + Fruquintinib:16.7 (95%CI: 11.1–22.3) months Fruquintinib: 14.6 (95%CI: 7.3–21.9) months, *p* = 0.120 ICI + Regorafenib: 14.1 (95%CI: 8.9–19.3) months Regorafenib: 15.7 months (95%CI: 9.8–21.6), *p* = 0.080 1-year OS: ICI + Fruquintinib: 62.2% Fruquintinib: 61.3%, *p* = 0.922 ICI + Regorafenib: 53.9% Regorafenib: 60.4%, *p* = 0.259 ORR: ICI + Fruquintinib:11.6% Fruquintinib: 4.3%, *p* = 0.097 ICI + Regorafenib: 4.9% Regorafenib: 6.0%, *p* > 0.999 DCR: ICI + Fruquintinib: 74.7% Fruquintinib: 60.0%, *p* = 0.044 ICI + Regorafenib: 70.4% Regorafenib: 61.2%, *p* = 0.240	None
Deng et al. (2023)	China	PD- 1 inhibitors (sintilimab, camrelizumab, tislelizumab, pembrolizumab, nivolumab, toripalimab) + Fruquintinib, *n* = 32 PD- 1 inhibitors (sintilimab, camrelizumab, tislelizumab, pembrolizumab, nivolumab, toripalimab) + Regorafenib, *n* = 27	Fruquintinib, *n* = 23 Regorafenib, *n* = 23	mPFS: ICI + Fruquintinib: 5.9 months Fruquintinib: 4.4 months, *p* = 0.009 ICI + Regorafenib: 3.8 months Regorafenib: 2.4 months, *p* = 0.262 mOS: ICI + Fruquintinib: 17.5 months Fruquintinib: 14.2 months, *p* = 0.008 ICI + Regorafenib: 14.8 months Regorafenib: 10 months, *p* = 0.045 ORR: ICI + Fruquintinib: 7.4% Fruquintinib: 4.5% ICI + Regorafenib: 3.7% Regorafenib: 0% DCR: ICI + Fruquintinib: 74.1% Fruquintinib: 54.5% ICI + Regorafenib: 59.3% Regorafenib: 47.6%	None
Wu et al. (2024)	China	PD- 1 inhibitors (camrelizumab, nivolumab, pembrolizumab, sintilimab, tislelizumab, toripalimab) + Fruquintinib, *n* = 29 PD- 1 inhibitors (camrelizumab, nivolumab, pembrolizumab, sintilimab, tislelizumab, toripalimab) + Regorafenib, *n* = 15	TAS- 102 + Bevacizumab, *n* = 16	mPFS: ICI + Fruquintinib: 4.2 months (95% CI: 2.1–5.2, *p* = 0.0113, HR = 0.41) ICI + Regorafenib: 6.3 months (95% CI: 3.6–9.0, *p* = 0.640, HR = 0.82) TAS- 102 + Bevacizumab: 5.4 months (95% CI: 0–12.5) ICI + Fruquintinib vs TAS- 102 + Bevacizumab: HR = 0.41, *p* = 0.0113 ICI + Regorafenib vs TAS- 102 + Bevacizumab: HR = 0.82, *p* = 0.6402 mOS: ICI + Fruquintinib: 16.0 months (95% CI: 10.2–21.8) ICI + Regorafenib: 19.2 months (95% CI: 9.1–29.3) TAS- 102 + Bevacizumab: 14.2 months (95% CI: 6.4–22.0), ICI + Fruquintinib vs TAS- 102 + Bevacizumab: HR = 0.89, *p* = 0.6646 ICI + Regorafenib vs TAS- 102 + Bevacizumab: HR = 0.50, *p* = 0.2994 ORR: ICI + Fruquintinib: 6.9% ICI + Regorafenib: 20% TAS- 102 + Bevacizumab: 0% DCR: ICI + Fruquintinib: 62.1% ICI + Regorafenib: 53.3% TAS- 102 + Bevacizumab: 78.6%	ICI + Fruquintinib group: Any grade TRAE: 48.3% Grade 3 + TRAE: 0% ICI + Regorafenib group: Any grade TRAE: 46.7% Grade 3 + TRAE: 13.3% TAS- 102 + bevacizumab group: Any grade TRAE: 43.8% Grade 3 + TRAE: 12.5%
Qu et al. (2024)	China	ICI (sintilimab, toripalimab, camrelizumab, tislelizumab, pembrolizumab) + Regorafenib, *n* = 161	Regorafenib, *n* = 376	mPFS: ICI + Regorafenib: 5.4 months Regorafenib: 3.8 months, *p* = 0.170 mOS: ICI + Regorafenib: 13.5 months Regorafenib: 10.0 months, *p* = 0.001	RI group: Any grade AE = 87.8% (*n* = 109) Grade 3 + AE = 13.7% (*n* = 12) R group: Any grade AE = 66.3% (*n* = 222) Grade 3 + AE = 15.2% (*n* = 51)
Wang et al. (2022)	China	PD- 1 inhibitors (drug not specified) + Regorafenib, *n* = 53	Regorafenib, *n* = 156	mOS: ICI + Regorafenib: 13.5 months Regorafenib: 10.1 months (95% CI: 0.325–0.879; HR = 0.534; *p* = 0.014) DCR: ICI + Regorafenib: 55% Regorafenib: 39%, *p* = 0.039	No statistical difference in TRAE between the two groups
Li et al. (2023)	China	ICI (sintilimab) + Fruquintinib, *n* = 47	Fruquintinib + TAS- 102, *n* = 45	mPFS: ICI + Fruquintinib: 6.0 months (95% CI: 4.589–7.411) Fruquintinib + TAS- 102: 3.5 months (95% CI: 0.964–6.145), *p* = 0.009 ORR: ICI + Fruquintinib: 17.0% Fruquintinib + TAS- 102: 13.3%, *p* = 0.623 DCR: ICI + Fruquintinib: 80.9% Fruquintinib + TAS- 102: 55.6%, *p* = 0.009	None
Li et al. (2024)	China	ICI (camrelizumab, tislelizumab, sintilimab) + TKI (regorafenib, fruquintinib), *n* = 40	TKI monotherapy (regorafenib, fruquintinib), *n* = 31	mPFS: ICI + TKI: 4.6 months (95% CI: 1.3–36.2) TKI monotherapy: 4.1 months (95% CI: 2.7–5.5), HR = 0.561, 95% CI: 0.34–0.94, *p* = 0.027 mOS: ICI + TKI: 15.8 months (95%CI: 7.3–24.3) TKI monotherapy: 13.2 months (95%CI: 9.9–16.4), HR = 0.671, 95% CI: 0.37–1.21, *p* = 0.189) ORR: ICI + TKI: 20.0% TKI monotherapy: 3.2%, OR = 0.080, 95% CI: 0.023–0.275, *p* = 0.000 DCR: ICI + TKI: 82.5% TKI monotherapy: 58.1%, OR = 0.024, 95% CI: 0.008–0.074, *p* = 0.000	No safety data reported
Gou et al. (2024)	China	ICI + TKI (regorafenib, fruquintinib), *n* = 51	Chemotherapy + bevacizumab, *n* = 62 TKI monotherapy, *n* = 16	mPFS: ICI + TKI: 3.5 months Chemotherapy + bevacizumab: 4.7 months TKI monotherapy: 2.2 months mOS: ICI + TKI: 10.3 months Chemotherapy + bevacizumab: 15.6 months TKI monotherapy: 8.4 months ICI + TKI vs Chemotherapy + bevacizumab vs TKI monotherapy: HR = 0.803, 95% CI: 0.662–0.972, *p* = 0.024	None
Zhao et al. (2024)	China	ICI monotherapy (camrelizumab, sintilimab, tislelizumab, toripalimab, pembrolizumab, penpulimab, nivolumab), *n* = 7	ICI + Chemotherapy, *n* = 19 ICI + TKI, *n* = 79 ICI + TKI + Chemotherapy, *n* = 23 ICI + anti-VEGF(R)/EGFR/HER2 mAb, *n* = 9 ICI + anti-VEGF(R) EGFR/HER2 mAb + Chemotherapy, *n* = 20	mPFS: ICI: 2.5 months (95% CI: 0.0–4.9) ICI + Chemotherapy: 4.0 months (95%CI: 1.0–7.0) ICI + TKI: 4.4 months (95%CI: 3.3–5.5) ICI + TKI + Chemotherapy: 3.5 months (95%CI: 0.1–6.8) ICI + anti-VEGF(R) EGFR/HER2 mAb: 4.0 months (95%CI: 0.0–8.3) ICI + anti-VEGF(R) EGFR/HER2 mAb + Chemotherapy: 4.4 months (95%CI: 0.0–8.0) mOS: ICI: 9.8 months (95%CI: 3.2–16.3) ICI + Chemotherapy: 10.6 months (95%CI: 7.3–13.8) ICI + TKI: 10.1 months (95%CI: 6.5–13.7) ICI + TKI + Chemotherapy: 14.3 months (95%CI: 4.6–24.1) ICI + anti-VEGF(R)/EGFR/HER2 mAb: 6.7 months (95%CI: 4.5–9.0) ICI + anti-VEGF(R) EGFR/HER2 mAb + Chemotherapy: 12.5 months (95%CI: 8.6–16.4)	Any grade AE: 93.7% Grade 3 + AE: 39.9% Any irAE: 42.0% Grade 3 + irAE: 13.3% The AEs of ICI-based therapy were consistent with those of ICIs, antiangiogenic agents or chemotherapy

Abbreviations: ICI, immune checkpoint inhibitor; MSI-H, high microsatellite instability; CRC, colorectal cancer; TTNT, time to next treatment; NR, not reached; IQR, interquartile range; AHR, adjusted hazard ration; CI, confidence interval; mPFS, median progression-free survival; mOS, median overall survival; MSS, microsatellite stable; TAS- 102, Trifluridine/Tipiracil; ORR, objective response rate; DCR, disease control rate; FP, fruquintinib + PD- 1 inhibitors; F, fruquintinib; R, regorafenib; RP, regorafenib + PD- 1 inhibitors; TRAE, treatment-related adverse events; AE, adverse events; ALT, alanine aminotransferase; AST, aspartate aminotransferase; RI, regorafenib + immune checkpoint inhibitor; TKI: tyrosine kinase inhibitor; VEGF(R), vascular endothelial growth factor (receptor); EGFR, epidermal growth factor receptor; HER2, human epidermal growth factor receptor 2; mAb, monoclonal antibody; irAE, immune-related adverse events.

### Immune Checkpoint Inhibitor Monotherapy

Only one study evaluated the efficacy of ICI monotherapy versus chemotherapy in mCRC [26]. The first cohort included 49 patients with MSI-H tumors who received first-line ICIs, including pembrolizumab or nivolumab monotherapy, or a combination of ipilimumab and nivolumab. This group was compared to 89 patients who received standard 5-fluorouracil (5-FU)-based chemotherapy [26]. Among patients with MSI-H tumors, ICIs demonstrated significantly improved outcomes compared to chemotherapy. The median PFS was 24.87 months in the ICI group, significantly surpassing the 5.65 months observed in the chemotherapy group (*p* < 0.001) with a hazard ratio (HR) of 0.31 (95% confidence interval [CI]: 0.18–0.52). Similarly, the median OS was not reached in the ICI group, indicating a sustained survival benefit, compared to 24.1 months in the chemotherapy group (*p* = 0.02), with a HR of 0.45 (95%CI: 0.23–0.88) [26]. Notably, the second cohort, consisting of 83 patients with MSI-H mCRC and 99 patients with MSS mCRC receiving ICI monotherapy at any line of therapy, showed more favorable outcomes for MSI-H compared to MSS tumors. The median PFS was 12.19 months in the MSI-H group versus 2.46 months in MSS group (p < 0.001). The median OS was not reached in the MSI-H group, compared to 6.57 months in the MSS group (p < 0.001) [26].

### Immune Checkpoint Inhibitor and Antineoplastic Agent Combination

Nie et al. assessed the efficacy of ICIs combined with trifluridine/tipiracil (TAS- 102) against TAS- 102 monotherapy and TAS- 102 with bevacizumab [27]. The combination of ICIs and TAS- 102 (*n* = 10) did not improve clinical outcomes compared to TAS- 102 alone (*n* = 6). The median PFS was identical in both groups at 3.0 months (ICI plus TAS- 102 combination: 95% CI: 1.5–4.5 and TAS- 102 monotherapy: 95% CI: 2.2–3.8; p = 0.041) [27]. Similarly, the median OS was 6.0 months for the ICI + TAS- 102 combination therapy (95% CI: 1.7–10.3) and 6.5 months for TAS- 102 monotherapy (95% CI: 0.1–12.9; p = 0.013). However, the DCR was lower in the ICI plus TAS- 102 combination group (40%) compared to 50% with TAS- 102 monotherapy (*p* = 0.047). The safety profile for ICI plus TAS- 102 combination therapy was comparable to TAS- 102 monotherapy, with no significant increase in TAS- 102-related adverse events [27].

### Immune Checkpoint Inhibitor and Tyrosine Kinase Inhibitor Combinations

A total of eight studies examined the efficacy of combining ICIs with tyrosine kinase inhibitors (TKIs) as the main treatment group for CRC, particularly in patients who had progressed after standard chemotherapy [28–35]. Regorafenib was the most commonly investigated TKI, featured in seven studies [28–33, 35]. While results were mixed, the overall findings suggested better efficacy outcomes with ICI-TKI combinations.

In a retrospective study by Wang et al., which included 209 mCRC patients, those receiving PD- 1 inhibitors plus regorafenib (*n* = 53) had a significantly longer median OS compared to those receiving regorafenib alone (*n* = 156) (13.5 vs. 10.1 months; *p* = 0.014) [28]. In a larger cohort study of 537 patients, the combination of ICI-regorafenib (*n* = 161) also resulted in a significantly longer median OS compared to regorafenib monotherapy (*n* = 376) (13.5 vs. 10.0 months; p = 0.001) [29]. However, the median PFS was 5.4 months for the ICI-regorafenib group, compared to 3.8 months for regorafenib monotherapy, with the difference not reaching statistical significance (p = 0.170) [29]. Similarly, Deng et al. found that patients treated with PD- 1 inhibitors plus regorafenib (n = 27) had a more prolonged median OS compared to regorafenib monotherapy (*n* = 23) (14.8 vs. 10.0 months; *p* = 0.045) [30]. In contrast, a study by An et al. involving 148 patients found no significant survival benefit from combining PD- 1 inhibitors (camrelizumab, sintilimab, tislelizumab, toripalimab, pembrolizumab or nivolumab) with regorafenib (*n* = 81) compared to regorafenib monotherapy (*n* = 67) for PFS, ORR, OS or DCR (*p* > 0.05) [31]. Similarly, when comparing the combination of ICI with regorafenib to TAS- 102 plus bevacizumab, Wu et al. found that although the ICI-regorafenib resulted in longer median PFS and median OS, the differences were not significant (*p* = 0.6402 for median PFS, and *p* = 0.2994 for median OS) [33].

Two studies examined the combination of PD- 1 inhibitors with fruquintinib, suggesting improved median PFS and DCR compared to fruquintinib monotherapy [30, 31]. The median PFS for the combination therapy ranged from 4.9 to 5.9 months, while monotherapy yielded a median PFS of 3.5 to 4.4 months [30, 31]. The DCR for the combination therapy ranged from 74.1% to 74.7%, compared to 54.5% to 60% for the monotherapy. Notably, Deng et al. reported a significant improvement in median PFS (5.9 vs. 4.4 months, *p* = 0.009) and in median OS with the combination therapy (17.5 vs. 14.2 months, *p* = 0.008) with the combination therapy [30]. These trends were also observed in the study by An et al. though they were not statistically significant [31]. Specifically, the combination therapy showed a longer median PFS (4.9 vs. 3.5 months) and longer median OS (16.7 vs 14.6 months), but the differences were not statistically significant (*p* > 0.05) [31]. Additionally, the ICI-fruquintinib combination group also showed significantly better survival benefits when compared to TAS- 102 plus fruquintinib, with improvements in median PFS and DCR (*p* < 0.05) [34]. Interestingly, one study found that the median PFS for the combination therapy was shorter than that for the TAS- 102 plus bevacizumab regimen (4.2 months vs. 5.4 months) [33]. Although the combination therapy resulted in longer median OS (16.0 months vs. 14.2 months), the improvement was not statistically significant (*p* > 0.05) [33].

Two studies compared the efficacy between the ICI-TKI combination therapy versus TKI monotherapy without stratifying by specific drugs [32, 35]. Both studies found longer median PFS and median OS in the combination therapy group, though the improvement were not statistically significant [32, 35]. Notably, Li et al. reported significantly better ORR and DCR in the 40 patients treated with ICI-TKI combination therapy (20% vs. 3.2% for ORR, 82.5% vs. 58.1% for DCR) [35].

### Immune Checkpoint Inhibitor Monotherapy and Other ICI-based Combinations

In a more recent real‐world study by Zhao et al. involving 143 heavily pretreated MSS mCRC patients, various ICI‐based combinations were assessed [36]. Patients received ICI-TKI combination therapy (TKIs included VEGFR inhibitors, BRAF inhibitors, MEK inhibitors, HER2 inhibitors and ALK inhibitors) yielded longer median PFS of 4.4 months (95% CI: 3.3–5.5) than those who received ICI monotherapy (2.5 months, 95% CI: 0.0–4.9) and ICI-Chemotherapy (4.0 months, 95% CI: 1.0–7.0) [36]. Additionally, ICI-TKI alone yielded a median OS of 10.1 months (95% CI: 6.5–13.7), while adding chemotherapy to ICI + TKI further prolonged OS to 14.3 months (95% CI: 4.6–24.1) [36]. In contrast, ICI monotherapy showed a shorter median OS of 9.8 months (95% CI: 3.2–16.3) [36]. These results suggest that combining ICI and TKI may offer additional survival benefits in MSS mCRC, especially when chemotherapy is also included.

### Safety Outcomes

Five of the 11 included studies compared safety outcomes between the ICI group and the comparator group [27–29, 33, 36]. Common adverse events (AEs) included hypertension, hand-foot skin reaction, and liver function abnormalities. The incidence of grade 3 or higher AEs was not significantly higher in patients receiving ICI-based regimens compared to those receiving regorafenib or fruquintinib monotherapy. For instance, Qu et al. reported any-grade AEs in 87.8% of patients receiving the ICI plus regorafenib combination, compared to 66.3% in those receiving regorafenib alone. However, the incidence of severe AEs was similar between the two groups (13.7% vs. 15.2%) [29]. Likewise, both Nie et al. and Wang et al. suggested no statistically significant difference in TRAE between the ICI-based regimens and the comparator group [27, 28].

### Quality Assessment

The quality assessment of the included studies is summarized in Supplementary Table S3. Overall, eight studies were classified as good quality [26, 29–32, 34–36]. These studies were deemed to have sufficient comparability between cohorts, as potential confounders were appropriately adjusted for in the multivariable Cox regression analyses [26, 29–32, 34–36]. In contrast, three studies were classified as poor quality due to the predominant use of univariate analysis without adjustment for confounders [27, 28, 33].

## Discussion

**Fig. 2:**
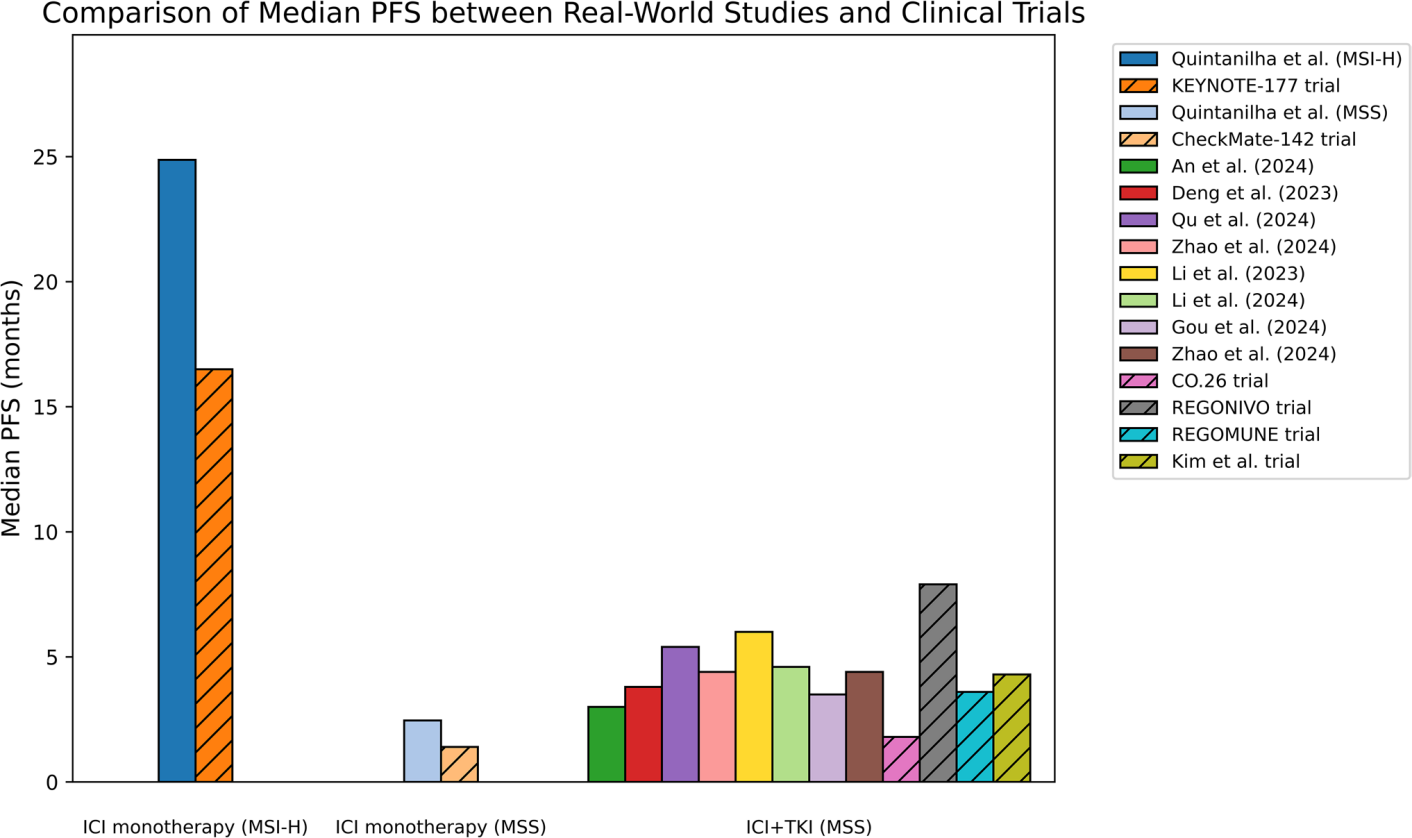
Median PFS (months) from high-quality real-world studies included in this review (represented by bars without hatch patterns) and previous clinical trials (represented by bars with hatch patterns), grouped by treatment modality. The first group (ICI Monotherapy (MSI-H)) shows data collected from MSI-H mCRC setting, with Quintanilha’s study (24.87 months) [26] and the KEYNOTE- 177 trial (16.5 months) [7]. The second group (ICI Monotherapy (MSS)) displays data from MSS mCRC setting, with Quintanilha’s study (2.46 months) and the CheckMate- 142 trial (1.4 months) [11, 26]. The third group (ICI + TKI (MSS)) included 12 studies conducted mainly in MSS mCRC setting—An et al. [31], Deng et al. [30], Qu et al. [29], Zhao et al. [36], Li et al. [34], Li et al. [35], Gou et al. [32], the CO.26 trial [38], the REGONIVO trial [13], the REGOMUNE trial [39], and Kim et al. trial [40], —with median PFS values ranging from 1.8 to 7.9 months

To the best of our knowledge, this is the first systematic review to integrate real-world observational evidence on the effectiveness and safety of ICIs in colorectal cancer. While ICIs have revolutionized the treatment of various solid tumors, their clinical utility in mCRC is currently limited to patients with dMMR/MSI-H, which account for only 5% of all CRC cases [7, 8, 12]. For example, the KEYNOTE- 177 trial, demonstrated that pembrolizumab significantly improves PFS compared to chemotherapy in patients with dMMR/MSI-H mCRC [7]. However, patients with pMMR/MSS disease have not shown similar benefits from ICI monotherapy, particularly in later lines of therapy [9–12]. The CheckMate- 142 trial, for example, reported a median PFS of only 1.4 months in non-MSI-H patients treated with nivolumab [11]. While these findings from clinical trials provide valuable insights, real-world evidence has become crucial in understanding the broader applicability of ICIs in mCRC treatment. One high-quality real-world study included in this review demonstrated that ICI monotherapy resulted in more favorable median PFS and median OS for patients with MSI-H tumors compared to those with MSS mCRC (Fig. 2) [26]. These findings align with clinical trial results, further supporting the efficacy of ICIs in the MSI-H population.

RCTs examining combination therapies, such as PD- 1 inhibitors plus targeted agents or chemotherapy, have shown favorable outcomes in both MSI-H and MSS mCRC compared to standard treatment [13, 39–44]. For example, in the MSI-H mCRC setting, a phase II trial involving 119 patients showed an ORR of 55% and a 12-months PFS of 71% [41]. In contrast, in MSS mCRC, the phase II REGOMUNE trial and a phase I/Ib trial by Kim et al. reported median PFS of 3.6 months and 4.3 months, respectively [39, 40]. The REGONIVO trial, which involved a combination of nivolumab and regorafenib, found a promising median PFS of 7.9 months, with the majority of participants having MSS disease (Fig. 2) [13]. This trial reported the longest median PFS seen in any study, which may be attributable to factors such as differences in study populations, treatment lines, and outcome measurements [13]. However, a limitation of many prior RCTs is their predominantly single-arm design, which makes it difficult to draw definitive conclusions about the superiority of combination strategies over standard-of-care regimens. The REGONIVO trial, for example, had stringent inclusion criteria, excluding individuals with serious comorbidities and those with Eastern Cooperative Oncology Group performance status (ECOG PS) of higher than 1 [13]. In contrast, real-world studies encompass a more diverse patient population, encompassing a broader range of ECOG PS (0 to 3), and patients with varying characteristics (e.g., ages spanning 18 to 88 years in real-world studies included in this review vs 31 to 77 years in the REGONIVO trial) [26–36]. Moreover, real-world studies tend to involve longer follow-up durations (10.8 to 28.4 months), providing a more comprehensive understanding of treatment efficacy and safety over time [26–36]. These factors highlight the complementary role of real-world studies in enhancing our understanding of treatment outcomes.

Real-world studies included in this review, which primarily reflect the MSS population, showed that combination therapies generally led to improved clinical outcomes compared to regorafenib monotherapy [28–30]. Specifically, the median OS with ICI plus regorafenib combination therapy in the three real-world studies included in this review was significantly longer than with regorafenib monotherapy in patients with MSS mCRC [28–30]. However, in two of the studies, the median PFS did not show significant improvement [29, 30]. Similar findings were observed in the phase II CO.26 trial [38], where the median OS of the ICI combination therapy was significantly better (6.6 months vs 4.1 months, *p* = 0.07), but the median PFS was 1.8 months in the ICI combination group compared to 1.9 months in the best supportive care group [34]. The limited improvement in PFS may be attributed to the nature of immunotherapy, which can extend survival even in the absence of clear tumor regression. Interestingly, one study found that combining a PD- 1 inhibitor with regorafenib did not provide a PFS advantage over regorafenib alone and was even associated with a higher incidence of adverse reactions [31]. A single-arm retrospective study also reported no ORR in any of the 23 MSS mCRC patients treated with the combination therapy [45]. Wu et al. similarly found no significant improvement in either median PFS or median OS when comparing the combination therapy to TAS- 102 plus bevacizumab [33]. However, the sample size was small (*n* = 60) and no clinical trials on this comparison were identified [33]. These findings underscore the need for further investigation, particularly with larger sample sizes, to better understand the potential of combination therapies in MSS mCRC and determine the most effective treatment strategies.

The combination of ICIs with fruquintinib, compared to fruquintinib monotherapy, has been explored in two real-world studies of patients with MSS mCRC, both of which reported more promising outcomes when ICIs were combined with fruquintinib [30, 31]. Specifically, these studies demonstrated superior median PFS, median OS, and DCR compared to fruquintinib monotherapy. Prior phase I clinical trials reported that the median PFS of the combination therapy ranged from 5.45 to 6.9 months [46–48], which aligns with the findings from Deng et al. [30]. Additionally, the efficacy of ICI combined with fruquintinib provided significantly longer median PFS compared to TAS- 102 plus fruquintinib [34]. These observations suggest a potentially synergistic effect, where the combination may alter the tumor microenvironment, thereby enhancing immune recognition [49].

The efficacy of ICI-TKI combination was also confirmed by a high-quality study that did not stratify for specific TKI agents. The ICI-TKI combination resulted in significantly higher mPFS, ORR and DCR compared to TKI monotherapy alone (*p* < 0.05) [35]. Further, in a direct comparison between ICI-TKI combination and ICI monotherapy, Zhao et al. found that the combination therapy achieved a longer median PFS and median OS in MSS mCRC patients (4.4 vs 2.5 months, 10.1 vs 9.8 months) [36]. This enhanced efficacy is likely driven by the anti-angiogenic effects of TKIs, which normalize tumor vasculature, improve perfusion and oxygenation, promote chemokine release, and enhance T cell infiltration, thereby creating a more immunosupportive microenvironment [50, 51].

Additionally, one real-world study reported no significant improvement in efficacy with the combination of ICIs and TAS- 102 [27]. However, no clinical trials have specifically evaluated this combination. Previous single-arm C-TASK FORCE trial only investigated TAS- 102 in combination with bevacizumab, either compared to TAS- 102 alone, or to regorafenib [52]. Therefore, further validation through large-scale studies is needed to better assess the efficacy of ICI and TAS- 102 combinations. 

Prior studies have suggested that liver metastasis may reduce the effectiveness of PD-1 inhibitors [53–55]. The REGOTORI study also showed a higher ORR in patients without liver metastases (30.0% vs 8.7%) [14]. Two studies included in this review specifically examined the impact of liver metastases on ICI efficacy in patients with MSS mCRC, consistently showing better clinical outcomes in patients without liver involvement [31, 36]. Additionally, Li et al. reported a higher median PFS in patients without liver metastases treated with an ICI-TKI combination [35]. These findings suggest that, despite the general resistance of MSS mCRC to ICIs, patients without liver metastases may still derive meaningful benefit from ICI-based therapies. ICI-based combination strategies may represent a promising approach to overcoming immunotherapy resistance and enhancing anti-tumor immune responses in this subgroup [55].

In terms of safety, five studies specifically compared the incidence of adverse effects between the ICI group and the comparator group [27–29, 33, 36]. Nie et al. and Wang et al. found no statistically significant differences in treatment-related adverse events between the ICI plus TAS- 102 combination and TAS- 102 monotherapy, and between PD- 1 inhibitors plus regorafenib and regorafenib monotherapy, respectively [27, 28]. Qu et al. reported comparable rates of Grade 3 + AEs between the ICI and regorafenib combination and regorafenib monotherapy [29]. Wu et al. found similar rate of treatment-related adverse events between ICI-TKI combination group and TAS- 102 plus bevacizumab group, ranging from 43.8% to 48.3% [33]. Only one study reported immune-related adverse events [36]. The incidence of any grade immune-related adverse events and grade 3–4 immune-related adverse events were higher in the real-world study compared to the KEYNOTE- 177 trial (42.0% vs 31%, 13.3% vs 9%) [7, 36]. This discrepancy likely reflects the broader, less-stringent patient population and the extended follow-up typical of real-world studies.

Compared to previous single-arm trials, the inclusion of comparator groups provides complementary evidence on the effectiveness and safety of ICIs relative to other treatments used in real-world settings. However, real-world observational studies are inherently subject to confounding and biases [26–36]. Eight studies addressed this issue by using multivariate adjustments, such as Cox regression models, to compare outcomes between cohorts [26, 29–32, 34–36]. Notably, Quintanilha et al., utilized inverse probability weighting to create a “pseudo-population” with balanced clinical risk across treatment groups [26]. Inverse probability weighting adjusts for confounders by assigning weights to each individual based on the inverse of their probability of receiving the treatment they actually received [56]. This approach reduces confounding by approximating a randomized setting, offering more reliable comparative survival estimates [26].

## Limitations

The strength of this review lies in its comprehensive search strategy, which utilized three electronic databases to capture real-world studies. However, a meta-analysis could not be conducted due to the heterogeneity among the included studies. The primary limitation is that only one study utilized electronic health records from multiple clinics in the US [26], while ten of the 11 studies were single-center, retrospective studies conducted in China [27–36], which may limit the generalizability of the findings. For instance, it is unclear whether the comparison drugs investigated were considered standard of care in other countries or regions. Notably, the proportion of CRC cases among Asians are lower than the global average [57], and Asians are less likely to have MSI-H tumors [58]. In addition, several studies involved a small number of patients with unknown MSI status [27–30], which may limit the ability to fully assess the efficacy of the drug therapies in these studies [27–30]. Overall, the studies included in this review had relatively small sample sizes, with the smallest involving just 10 people in the ICI plus TAS- 102 combination therapy group and 6 people in the TAS- 102 group [27], and the largest involving 161 people in the ICI-regorafenib combination therapy and 376 people in the regorafenib group [29]. Larger real-world studies are needed to better understand the comparative effectiveness and safety of ICIs in CRC.

## Conclusion

This review is one of the first to examine the real-world comparative effectiveness and safety of ICIs in patients with CRC. The current evidence base is limited, with only 11 small-scale studies conducted, predominantly conducted in China. Based on the available data, our systematic review highlights the potential of ICIs in the treatment of mCRC, particularly in the MSI-H/dMMR subset, where ICI monotherapy demonstrates clinical benefit. For the more common MSS/pMMR mCRC, combining ICIs with TKIs like regorafenib or fruquintinib, or with chemotherapy, shows promise, although additional reliable data are needed. Only five of the 11 included studies assessed the safety of ICIs against comparator groups, and they suggest similar safety profiles between the regimens. Given the limited number of real-world studies to date and the heterogeneity of patient populations and treatment regimens in the available studies, further well-designed, large-scale studies are needed. Such studies should aim to confirm the effectiveness and safety of ICI-based combination therapies in CRC and provide a clearer understanding of their role in clinical practice.

## Key References

- Quintanilha, J. C. F. et al*.* Comparative Effectiveness of Immune Checkpoint Inhibitors vs Chemotherapy in Patients With Metastatic Colorectal Cancer With Measures of Microsatellite Instability, Mismatch Repair, or Tumor Mutational Burden. *JAMA Netw. Open* 6, e2252244 (2023). https://doi.org/10.1001/jamanetworkopen.2022.52244.
  - This real-world study conducted in US includes two cohorts, the ICI monotherapy group shows a PFS of 24.87 months in MSI-H mCRC patients, while showing limited benefit in MSS mCRC patients.
- Qu, W. et al*.* Regorafenib monotherapy or combined with an immune-checkpoint inhibitor as later-line treatment for metastatic colorectal cancer: a multicenter, real-world retrospective study in China. *BMC Cancer* **24**, 22 (2024). https://doi.org/10.1186/s12885-023–11700-w.
  - This real-world study includes the largest cohort of mCRC patients and shows promising effectiveness with ICI-TKI combination therapy.
- Deng, Y.-Y. et al*.* Comparison of the efficacy and safety of fruquintinib and regorafenib in the treatment of metastatic colorectal cancer: A real-world study. *Front. Oncol.* **13**, 1097911 (2023). https://doi.org/10.3389/fonc.2023.1097911.
  - This real-world study on mCRC shows PD- 1 inhibitors + fruquintinib results in significantly longer mPFS and mOS than fruquintinib alone.
- An, T.-Q. et al*.* Efficacy comparison of fruquintinib, regorafenib monotherapy or plus programmed death- 1 inhibitors for microsatellite stable metastatic colorectal cancer. *World J. Gastrointest. Oncol.* **16**, 2449–2462 (2024). https://doi.org/10.4251/wjgo.v16.i6.2449.
  - This real-world study brings interesting results on 313 MSS mCRC patients. The PD- 1 inhibitors + fruquintinib combination is superior to fruquintinib alone in achieving 6-months PFS, while PD- 1 inhibitors + regorafenib combination does not show better effectiveness than regorafenib alone.
- Gou, M., Qian, N., Zhang, Y. et al*.* Third- or Further-Line Treatment in Patients with MSS Type Metastatic Colorectal Cancer. *J Gastrointest Canc* **56**, 21 (2025). https://doi.org/10.1007/s12029-024–01120-9.
  - This real-world study on 129 patients shows longer mPFS in the ICI-TKI combination group than TKI alone without significant differences.
- Li L, Wang T, Wu Z, Li Y, Ma H, Wang L, Lei S, Chen W. Fruquintinib in combination with sintilimab or TAS- 102 as third-line or above treatment in patients with metastatic colorectal cancer: a real-world study. *Transl Cancer Res*. 12(11):3034 - 3044 (2023). https://doi.org/10.21037/tcr-23–867.
  - This real-world study shows ICI-fruquintinib combination provides longer mPFS than fruquintinib + chemotherapy in MSS mCRC.
- Li D, Jin H, Liu Y, Liu J, Zhang X, Wang L, Fan Z, Feng L, Zuo J, Han J and Wang Y. Identification of beneficial populations for targeted-immunotherapy combinations: tailoring later-line care for patients with pMMR/MSS metastatic colorectal cancer. *Front. Immunol*. 15:1462346 (2024). https://doi.org/10.3389/fimmu.2024.1462346.
  - This is the most recently published study comparing ICI-TKI combination and TKI monotherapy in MSS mCRC.
- Zhao, W. & Chen, Y. Efficacy and safety of immune checkpoint inhibitors in heavily pretreated patients with microsatellite stable metastatic colorectal cancer: a real-world retrospective study. *Am. J. Cancer Res.* **14**, 5378–5388 (2024). https://doi.org/10.62347/KAFY8529.
  - To date, this is the only real-world study that compares multiple ICI-based regimens for MSS mCRC patients.

## Supplementary Information

Below is the link to the electronic supplementary material.

Supplementary file1 (DOCX 26.4 KB)

## Data Availability

No datasets were generated or analysed during the current study.
