# Supplementary material for: Effectiveness and Safety of Immune Checkpoint Inhibitors in Colorectal Cancer: A Systematic Review of Real-World Studies
Source: Curr Oncol Rep. 2025 May 13;27(6):687–702. doi: 10.1007/s11912-025-01676-0 (PMC12227369; doi:10.1007/s11912-025-01676-0)
Supplement: Supplementary file 1 — Supplementary file1 (DOCX 26.4 KB) [file 11912_2025_1676_MOESM1_ESM.docx]

**Article title:**

Effectiveness and Safety of Immune Checkpoint Inhibitors in Colorectal Cancer: A Systematic Review of Real-World Studies

**Journal name:**

Current Oncology Reports

**Author information**

Leping Kong,^1,2^ Chin Hang Yiu,^1,2^ Christine Y. Lu (corresponding author)^1,2,3^

**Affiliations:**

^1^ The University of Sydney School of Pharmacy, Camperdown, New South Wales, Australia

^2^ Kolling Institute, Faculty of Medicine and Health, The University of Sydney and the Northern Sydney Local Health District, Sydney, New South Wales, Australia

^3^ Department of Pharmacy, Royal North Shore Hospital, St Leonards, New South Wales, Australia

**Corresponding author:**

Prof Christine Lu

christine.lu@sydney.edu.au

**Supplementary Table S1: Changes to PROSPERO protocol with reasons**

| **Number** | **Protocol** | **Change(s)** | **Reason(s)** |
| --- | --- | --- | --- |
| **1** | Search was done from database inception to 31^st^ August 2024. | Search was done from database inception to March 15, 2025. | To ensure that the search is up to date, no newly published articles are missing. |
| **2** | Search was done through four databases: PubMed, MEDLINE, Embase and Scopus. | Search was done through three databases MEDLINE, Embase and Scopus. | Scopus, Embase and Medline were used (Medline is the largest subset of PubMed). |
| **3** | The relevant efficacy endpoints included complete response rate (CR), partial response rate (PR), stable disease rate (SD), etc. | Complete response rate (CR), partial response rate (PR), and stable disease rate (SD) were not reported by studies included in this review, and therefore, were not recorded. | These efficacy outcomes were not specifically reported in articles included in this review. Instead, objective response rate (ORR) was used and reported by the studies. |
| **4** | Efficacy and safety outcomes were categorized into “Main outcomes” and “Additional outcomes”. | Both efficacy and safety outcomes were main outcomes of this review. | We acknowledged that safety outcomes in real-worlds studies, which capture diverse patient populations are crucial. We also recognized the importance of balancing clinical benefits with potential risks. |
| **5** | A meta-analysis was planned. | A meta-analysis was not conducted. | Data synthesis was not conducted due to the high heterogeneity in study designs, populations, outcome measures, and statistical approaches. |

**Supplementary Table S2: Search strategy and search results**

1. **Embase**

| Number | Query | Number of results |
| --- | --- | --- |
| 1 | colorectal cancer.mp. or colorectal cancer/ | 290510 |
| 2 | colorectal carcinoma.mp. or colorectal carcinoma/ | 38898 |
| 3 | immune checkpoint inhibitor/ | 40455 |
| 4 | ("immune checkpoint" or "Ipilimumab" or "atezolizumab" or "camrelizumab" or "sintilimab" or "tislelizumab" or "toripalimab" or "pembrolizumab" or "penpulimab" or "nivolumab").mp. | 151209 |
| 5 | medical record/ or electronic medical record/ | 309737 |
| 6 | ("claim*" or "administrative claim" or "routine clinical" or "hospital" or "administrative data" or "claim*data*" or "prescription data" or "health*data*" or "health system*" or "medical record*" or "medical chart*" or "electronic health record*" or "real world" or "seer" or "medicare" or "insurance" or "comparative effectiveness").mp. | 4333304 |
| 7 | comparative effectiveness/ | 154781 |
| 8 | ("efficacy" or "treatment outcome*" or "clinical outcome*" or "survival" or "treatment response" or "safety").mp. | 6680975 |
| 9 | 1 or 2 | 312620 |
| 10 | 3 or 4 | 151209 |
| 11 | 5 or 6 | 4333304 |
| 12 | 7 or 8 | 6723119 |
| 13 | 9 and 10 and 11 and 12 | 649 |

1. **MEDLINE**

| Number | Query | Number of results |
| --- | --- | --- |
| 1 | colorectal cancer.mp. or colorectal cancer/ | 181782 |
| 2 | colorectal carcinoma.mp. or colorectal carcinoma/ | 128030 |
| 3 | 1 or 2 | 186739 |
| 4 | immune checkpoint inhibitor/ | 14195 |
| 5 | ("immune checkpoint" or "Ipilimumab" or "atezolizumab" or "camrelizumab" or "sintilimab" or "tislelizumab" or "toripalimab" or "pembrolizumab" or "penpulimab" or "nivolumab").mp. | 69369 |
| 6 | 4 or 5 | 97205 |
| 7 | medical record/ or electronic medical record/ | 2388096 |
| 8 | ("claim*" or "administrative claim" or "routine clinical" or "hospital" or "administrative data" or "claim*data*" or "prescription data" or "health*data*" or "health system*" or "medical record*" or "medical chart*" or "electronic health record*" or "real world" or "seer" or "medicare" or "insurance" or "comparative effectiveness").mp. | 2388096 |
| 9 | 7 or 8 | 2388096 |
| 10 | comparative effectiveness/ | 4114 |
| 11 | ("efficacy" or "treatment outcome*" or "clinical outcome*" or "survival" or "treatment response" or "safety").mp. | 4276122 |
| 12 | 10 or 11 | 4278534 |
| 13 | 3 and 6 and 9 and 12 | 96 |

1. **Scopus**

| Query | Number of results |
| --- | --- |
| TITLE-ABS-KEY ( ( "colorectal cancer" OR "colorectal carcinoma" ) AND ( "immune checkpoint" OR "Ipilimumab" OR "atezolizumab" OR "camrelizumab" OR "sintilimab" OR "tislelizumab" OR "toripalimab" OR "pembrolizumab" OR "penpulimab" OR "nivolumab" ) AND ( "claim*" OR "administrative claim*" OR "routine clinical" OR "hospital" OR "administrative data*" OR "claim*data*" OR "prescription data*" OR "health*data*" OR "health system*" OR "medical record*" OR "medical chart*" OR "electronic health record*" OR "real world" OR "seer" OR "medicare" OR "insurance" ) AND ( "efficacy" OR "treatment outcome*" OR "clinical outcome*" OR "survival" OR "treatment response*" OR "safety" ) ) | 358 |

**Supplementary Table S3: Newcastle-Ottawa Quality Assessment for cohort studies (n=11)**

| **Author (Year)** | **Selection** | | | | **Comparability** | **Outcome** | | | **Quality**  **(AHRQ standards)** |
| --- | --- | --- | --- | --- | --- | --- | --- | --- | --- |
|  | Representativeness of exposed cohort  (Max: 1★) | Selection of the non-exposed cohort  (Max: 1★) | Ascertainment of exposure  (Max: 1★) | Demonstration that outcome of interest was not present at start of study  (Max: 1★) | Comparability of cohorts on the basis of design and analysis  (Max: 2★) | Assessment of outcome  (Max: 1★) | Was follow-up long enough for outcomes to occur^†^  (Max: 1★) | Adequacy of follow-up cohorts  (Max: 1★) |  |
| Quintanilha et al. (2023) | ★ | ★ | ★ | ★ | ★★  Multivariate Cox proportional hazards regression models were used to adjust for potential confounders across all analyses. Inverse probability weighting was used to help account for differences in baseline risk between groups. | ★ | ★  Median duration of follow-up: 20.66 months | ★ | Good |
| Nie et al. (2023) | ★ | ★ | ★ | ★ | –  No confounders were adjusted. Only baseline characteristics between cohorts were reported. | ★ | –  Median duration of follow-up not reported | ★ | Poor |
| An et al. (2024) | ★ | ★ | ★ | ★ | ★★  Multivariate Cox proportional hazards regression models were used to adjust for potential confounders across all analyses. | ★ | ★  Median duration of follow-up: 18.8 months | ★ | Good |
| Deng et al. (2023) | ★ | ★ | ★ | ★ | ★★  Multivariate Cox proportional hazards regression models were used to adjust for potential confounders across all analyses. | ★ | –  Median duration of follow-up not reported | ★ | Good |
| Wu et al. (2024) | ★ | ★ | ★ | ★ | –  Only univariate Cox regression analysis was used | ★ | ★  Median duration of follow-up: 12.6 months | ★ | Poor |
| Qu et al. (2024) | ★ | ★ | ★ | ★ | ★★  Multivariate Cox proportional hazards regression models were used to adjust for potential confounders across all analyses. | ★ | ★  Median duration of follow-up: 28.4 months | ★ | Good |
| Wang et al. (2022) | ★ | ★ | ★ | ★ | –  Cox proportional hazards regression models were used to analyse prognostic factors for overall survival only. | ★ | –  Median duration of follow-up: 11.3 months | ★ | Poor |
| Li et al. (2023) | ★ | ★ | ★ | ★ | ★★  Multivariate Cox proportional hazards regression models were used to adjust for potential confounders across all analyses | ★ | ★  Median duration of follow-up: 10.79 months | ★ | Good |
| Li et al. (2024) | ★ | ★ | ★ | ★ | ★★  Multivariate Cox proportional hazards regression models were used to adjust for potential confounders across all analyses | ★ | –  Median duration of follow-up not reported | ★ | Good |
| Gou et al. (2024) | ★ | ★ | ★ | ★ | ★★  Multivariate Cox proportional hazards regression models were used to adjust for potential confounders across all analyses | ★ | –  Median duration of follow-up not reported | ★ | Good |
| Zhao et al. (2024) | ★ | ★ | ★ | ★ | ★★  Multivariate Cox proportional hazards regression models were used to adjust for potential confounders across all analyses. | ★ | ★  Median duration of follow-up: 23.1 months | ★ | Good |

^†^ Acceptable length of follow-up = 12 months

Good quality: 3 or 4 stars in selection domain AND 1 or 2 stars in comparability domain AND 2 or 3 stars in outcome/exposure domain

Fair quality: 2 stars in selection domain AND 1 or 2 stars in comparability domain AND 2 or 3 stars in outcome/exposure domain

Poor quality: 0 or 1 star in selection domain OR 0 stars in comparability domain OR 0 or 1 stars in outcome/exposure domain

Abbreviations: AHRQ, Agency for Healthcare Research and Quality
